# Iranian psychiatrists’ attitude towards clozapine use for patients with treatment-resistant schizophrenia: a nationwide survey

**DOI:** 10.1186/s12888-022-04179-5

**Published:** 2022-08-06

**Authors:** Leeba Rezaie, Azadeh Nazari, Roya Safari-Faramani, Shamarina Shohaimi, Habibolah Khazaie

**Affiliations:** 1 Sleep Disorders Research Center, Kermanshah University of Medical Sciences, Kermanshah, Iran; 2 Student Research Committee, Kermanshah University of Medical Sciences, Kermanshah, Iran; 3 Department of Epidemiology, School of Health Social Development and Health Promotion Research Center Research Institute for Health, Kermanshah University of Medical Sciences, Kermanshah, Iran; 4 Department of Biology, Faculty of Science, Universiti Putra Malaysia, Serdang, Selangor Malaysia

**Keywords:** Clozapine, Treatment-resistant schizophrenia, Iran

## Abstract

**Background:**

Clozapine has the greatest efficacy for treatment-resistant schizophrenia (TRS), even though its underutilization is not uncommon across different countries. This study aimed to investigate the knowledge and attitude of Iranian psychiatrists toward clozapine use.

**Method:**

In this cross-sectional study, a questionnaire was distributed among psychiatrists registered with the Iranian Psychiatrists Association (including its provincial branches) to assess their knowledge and attitude towards clozapine use. A total of 282 psychiatrists completed the questionnaire. Descriptive analysis was used to describe demographic information, and Chi-square tests were conducted to determine if there is an association between academic position and work experience. All statistical analyses were performed using SPSS® version 25.0 for Windows, and a significance level of 0.05 was used.

**Results:**

Most respondents (93%) acknowledged that they prescribed clozapine for their patients, and 74% believed that clozapine was more effective than other antipsychotic drugs. However, 43.3% of the respondents said they did not believe in the safety of clozapine. Difficulty initiating and having no firsthand experience in the superiority of clozapine were reported by 81.2 and 80% of the respondents, respectively. Our results also showed an association between having an academic position and access to appropriate facilities for the control and management of patients treated with clozapine and believing in the safety of clozapine (*p* < 0.05). Longer work experience (more than 15 years) was associated with a higher prescription of clozapine, belief in greater effectiveness of clozapine, and its safety (*p* < 0.0001).

**Conclusion:**

Iranian psychiatrists had a good self-perception of knowledge about the efficacy of clozapine for patients with TRS, but concerns about serious side effects are common. Psychiatrists with longer work experience and academic positions were more optimistic towards clozapine use than the younger ones with no academic position. Considering the results in planning the strategies to decrease concerns about clozapine use is recommended.

## Introduction

Treatment-resistant schizophrenia (TRS) is defined as nonresponse to at least two trials of antipsychotic medications and is a serious condition for psychiatrists to manage [1, 2]. Patients with TRS usually suffer from more severe psychopathology, more impaired cognitive function, and weaker psychosocial adjustment compared to other patients with schizophrenia which causes more burden of disease [3]. It is estimated that TRS affects 20-30% of patients with schizophrenia [4]. Clozapine, an atypical antipsychotic drug, was known as an effective treatment for TRS in 1998, and since then, it has been used worldwide [5]. After the initial use, the efficacy of clozapine has been reported in several studies, including a meta-analysis by Davis and colleagues (2003) and Essali and colleagues (2009), a review by Agid and colleagues (2010), and a meta-analysis by Leuchtand and colleagues (2013, [6–9]). It is also reported that treatment initiation with clozapine decreases the re-hospitalization rate [10]. Therefore, it is considered the first line of treatment for TRS. However, the concern about some adverse side effects is also underlined in several reports. Neutropenia, agranulocytosis, seizures, QTc-prolongation, and myocarditis are some of the reported life-threatening side effects of clozapine. Because of the risk of neutropenia, and agranulocytosis, the systematic approach of white blood cell count (WBC) and absolute neutrophil count (ANC) monitoring before initiating and periodically thereafter initiating is recommended in the clinical guidelines [11, 12]. On the other hand, other side effects, including hypersalivation, drowsiness, constipation, hypotension, weight gain, and tachycardia, may impact drug tolerability. Consequently, patients’ poor adherence and discontinuation should not be overlooked [13].

Despite the growing body of evidence of the superiority of clozapine for the treatment of patients with TRS, there are several reports about limited access of the patients to treatment with clozapine, long delay to initiate, and underutilization of clozapine in different countries [14–17]. The mean 47.4 months of delay in initiating clozapine and polypharmacy and high–dose treatment with other antipsychotics have been reported in the study by Howes and colleagues (2012) [18]. Wheeler in New Zealand reported almost 10 years of theoretical delay in initiating clozapine [19]. The delay and underutilization may lead to poorer outcomes and, consequently, poorer quality of life in patients with TRS [16]. Previous studies showed several reasons for the underutilization of clozapine for patients with TRS. In a systematic review, Farooq et al. (2019) classified the barriers to clozapine use into three groups barriers related to patients and the drug; clinician-related barriers; and health system-related factors [20]. The first group addressed refusal from a mandatory blood test and drug tolerability. In the second group of barriers, inadequate knowledge and experience of clinicians in using clozapine and fear of life-threatening side effects, especially agranulocytosis, were among the related barriers. Finally, the last group of health system-related barriers included service fragmentation and inadequate resource for admission of TRS patients to initiate clozapine. While the systematic review could integrate the results of the studies to explore the barriers to clozapine use for patients with TRS, since the psychiatrists usually have the key role in selecting eligible patients, initiating clozapine, and following the patients, their attitude toward initiating the process of clozapine use and manage the potential barriers is important. The attitude of psychiatrists has been investigated in several studies. Tungaraza and Farooq (2015), in their research to assess the attitude of UK psychiatrists, reported that 40.5% of the respondent of the survey preferred to use other antipsychotics prior to initiating clozapine, and a third of them did not think clozapine use in the community is safe. Also, 42.7% did not feel clozapine can reduce the risk of substance use. The authors emphasized that concern about managing side effects was important and considered in the training program for psychiatrists [21]. In a similar study, Daod et al. in Israel (2019) reported that clozapine was used according to the guideline by 53% of psychiatrists, whereas 35% of them initiated clozapine use only after three or more unsuccessful trials of other antipsychotics. These authors also reported concern about side effects delaying using clozapine in eligible patients [22].

Iran, the focus of this study, is the second-largest country in the Middle East, with a population of more than 80 million. Based on a population-based study, the prevalence of people suspected of mental disorders in Iran is 23.44%, while the prevalence is higher in urban than rural areas (24.55 and 20.89%, respectively [23]). Meanwhile, there has been a dramatic improvement in primary health care and education infrastructure in the last two decades. School mental health (for example, life skills and parenting skills at schools), suicide prevention programs, psychosocial interventions for survivors of natural disasters, and shifting from long-term care of mental disorders to daycare are among the improvements that have been achieved [24]. Also, in rural areas, mental health care is integrated into the primary health care system. In this program, primary mental health is generally delivered by general practitioners and multipurpose community health workers named “behvarz” [25, 26]. The program aimed to increase access to mental health services, promote mental health services in local culture, and increase public awareness of mental disorders [27]. However, there is concern about the inadequacy of mental health services due to continued industrialization and urbanization [28]. With regard to clozapine, it is used for eligible patients according to evidence-based clinical guidelines [29]. Accordingly, when a patient with schizophrenia does not respond to trials of antipsychotics, each taken with adequate dose and duration (2 trials of ≥6 weeks), they were diagnosed with TRS. The psychiatrist who assessed them initiated clozapine. It should be mentioned that there are no specific centers for clozapine prescribing in Iran, and eligible patients usually are admitted to psychiatric wards to initiate clozapine prescribing. However, there may be outpatient’s initiation from some psychiatrists. While clozapine is not an out-pocket treatment in Iran, the periodic necessary lab tests and the need for regular appointments with psychiatrists, especially for patients who should travel to the larger cities to get the services, may be associated with financial difficulty for some patients. Therefore, psychiatrists may pay attention to the difficulty when they want to initiate clozapine. However, there is no formal report about the delay time to initiate clozapine due to the likelihood of barriers. Also, to the best of our knowledge, there is no study investigating Iranian psychiatrists’ views toward clozapine use for patients with TRS. Therefore, the study aimed to examine Iranian psychiatrists’ views about clozapine use for patients with TRS.

## Methods

### Study design

This is a nationwide cross-sectional study conducted in 2019 on Iranian psychiatrists.

### Study participants

Study participants were Iranian psychiatrists working at the time of study and were willing to participate in the study. They were approached via the Iranian Psychiatric Association (IPA) across the different provinces. Registered psychiatrists whose addresses and contact information were available in the provincial branches were included. Psychiatrists who did not complete the questionnaires were excluded from the study.

### Sample size

The standard formula: *n* = P × (1- P) × z^2^ /d^2^ was used to determine the required sample size. After adjusting for nonresponse, we calculated the minimum sample size of this study as 306 psychiatrists. The proportion was based on Davod et al. [22] and 11% precision and a confidence level of 95%.

### Data collection

The questionnaire was adapted from Tungaraza and Farooq (2015) with the authors’ permission. It had three main parts. The first part addressed the participants’ demographic information (e.g., their working experiences). The second part contained questions asking about the service characteristics of clozapine use; most of them were answered by yes and no. The last part deals with the knowledge, attitude, and experience of clozapine use. The questions were closed into five options using a Likert scale ranging from completely disagree, disagree, don’t know, agree, and completely agree [21].

To achieve sound psychometric properties in the Iranian context, the English version of the questionnaire was independently translated into Persian by two fluent English speakers and then back-translated into English by an independent English speaker. The Persian version of the questionnaire was sent to ten psychiatrists and asked about their opinion about the questionnaire’s content to check the content validity. All the psychiatrists acknowledged using a questionnaire to assess the attitude of psychiatrists on the prescription of clozapine.

Internal consistency of the questionnaire was assessed using Cronbach’s alpha. Kaiser-Meyer-Olkin was statistically significant (*p* < 0.0001), indicating sampling adequacy. Cronbach’s alpha was 0.93, which is considered satisfactory. The intraclass correlation coefficient (ICC) was used to assess the test-retest reliability of the questionnaire, which was ICC = 0.882, indicating excellent inter-rater reliability.

After coordination with the provincial branches of IPA, a printed questionnaire was posted to psychiatrists. The psychiatrists who desired to participate in the study filled out the questionnaire and mailed it back. All returned questionnaires were checked, and incomplete questionnaires were excluded.

### Statistical analysis

We used descriptive statistics to describe demographic information and the view of the participants. Responses to the third part of the questionnaire were analyzed by frequency and categorized into “agree,” “don’t know,” and “disagree.” Responses of “agree” and “fully agree” were categorized as “agree.” Responses of “fully disagree” and “disagree” were categorized as “disagree.” Chi-square tests were conducted to determine if significant differences in agreement to individual items on the questionnaire existed based on years of work experience (< 15 years, and ≥ 15 years) and an academic position (to have and not to have). All statistical calculations were performed with SPSS® version 25.0 for Windows, and a significance level of 0.05 was used.

## Results

### Demographic characteristics of participants

In total, 282 psychiatrists (response rate 92.2%) completed the questionnaire. Of these, 56.4% were male, and the mean (standard deviation) was 48.1 (8.4) years. About 18% of the participants had an academic position, and more than a third of them had more than 10 years of experience as faculty members. The mean (standard deviation) duration of activity as a psychiatrist was 14.4 (8.6) years. The demographic characteristics of the participants are presented in Table 1.

**Table 1:** Demographic characteristics of the psychiatrists participating in the study

Variable	Frequency (%)
Gender	
Female	122 (43.6)
Male	158 (56.4)
Age groups	
31-40 years old	58 (20.9)
41–50 years old	113 (40.8)
51 and more	106 (38.3)
Academic position	
Yes	48 (17.5)
No	226 (82.5)
Years of having an academic position	
Less than ten years	21 (36.8)
10-20 years	21 (36.8)
20 years and more	15 (26.3)
Mean (SD) of having an academic position	13.6 (7.9)
Years of working as a Psychiatrist	
Less than 15 years	144 (52.2)
15 years and more	132 (47.8)

### Service characteristics of using clozapine

The service characteristics of using clozapine are presented in Table 2. About 65% of participants reported a home visit team and crisis intervention in the organization. However, over 90% reported that they do not have access to dedicated physicians for clozapine use in the organization. About 93% of the participants reported prescribing clozapine during their career, and about 69% reported having at least five patients treated with clozapine. Less than half a percent of participants experienced serious events related to clozapine administration in the past 6 months. More than 80% have read articles related to clozapine during their career.

**Table 2:** Services characteristics of using clozapine

	Category	Frequency (percent)
Crisis/home treatment team	Yes	182 (64.8)
	No	99 (35.2)
Having a physician leading a dedicated clozapine team in the organization	Yes	15 (5.5)
	No	260 (94.5)
Prescribing of clozapine during the working period	Yes	280 (92.8)
	No	20 (7.2)
Prescribing of clozapine in the last three months	Yes	73 (28.4)
	No	184 (71.6)
Prescribing of clozapine in the last six months	Yes	67 (26.1)
	No	190 (73.9)
Prescribing of clozapine in the last year	Yes	79 (30.9)
	No	177 (69.1)
Number of patients under the treatment with clozapine	Less than five patients	175 (68.9)
	5-10 patients	62 (24.4)
	10 and more	17 (6.7)
Mean (SD) patients under the treatment with clozapine		4.3 (6.7)
Experiencing serious events related to clozapine over the past six months	Yes	1 (0.4)
	No	261 (99.60
Experiencing serious events related to clozapine over the last year	Yes	0
	No	261 (100)
Experience serious events related to clozapine sometimes in the past	Yes	7 (2.7)
	No	256 (97.3)
The negative effect of side effects on attitude towards clozapine	Yes	2 (7.7)
	No	24 (92.3)
Study article on clozapine during the work period	Yes	226 (80.7)
	No	54 (19.3)
Study article on clozapine in the past three months	Yes	75 (100)
	No	0
Study article on clozapine in the past six months	Yes	147 (100)
	No	0

### Knowledge, attitude, and experience in the use of clozapine

Our results showed that about 94% of psychiatrists in the study started clozapine for their patients, and 74% believed that clozapine was more effective than other antipsychotic drugs. About half of the psychiatrists had access to appropriate facilities for the control and management of patients treated with clozapine, and 43.3% did not know of the safety of clozapine. Most respondents (73%) use other strategies before starting clozapine when treatment fails with 2 or 3 antipsychotics. Almost all participants (97%) stated that they had access to counseling services if they were concerned about a patient being treated with clozapine, and 81.2% agreed with the difficulty of initiating clozapine treatment. Thirty-five percent (35%) of participants believed they could not judge its effectiveness due to controversies about clozapine, while 40% agreed that clozapine-related life-threatening side effects discouraged them from prescribing it. Sixty percent (60%) of respondents disagreed with concerns about increasing the likelihood of drug interactions, and 63% believed there was a limit to the number of patients for whom clozapine could be started due to financial issues. Seventy-two percent (72%) disagreed that clozapine increased the risk of death compared to other antipsychotic drugs. Fifty-five percent (55%) felt “clozapine should be delayed due to the lack of other suitable alternatives.” Sixty percent (60%) believed it is not easy to quickly diagnose patients who benefit from changing their medication to clozapine. Sixty-four percent (64%) disagreed with the safety of patients starting treatment with clozapine, while 45 % (45%) of the psychiatrists in the study believed that clozapine was associated with reducing alcohol and drug use among patients treated.

**Fig. 1:**
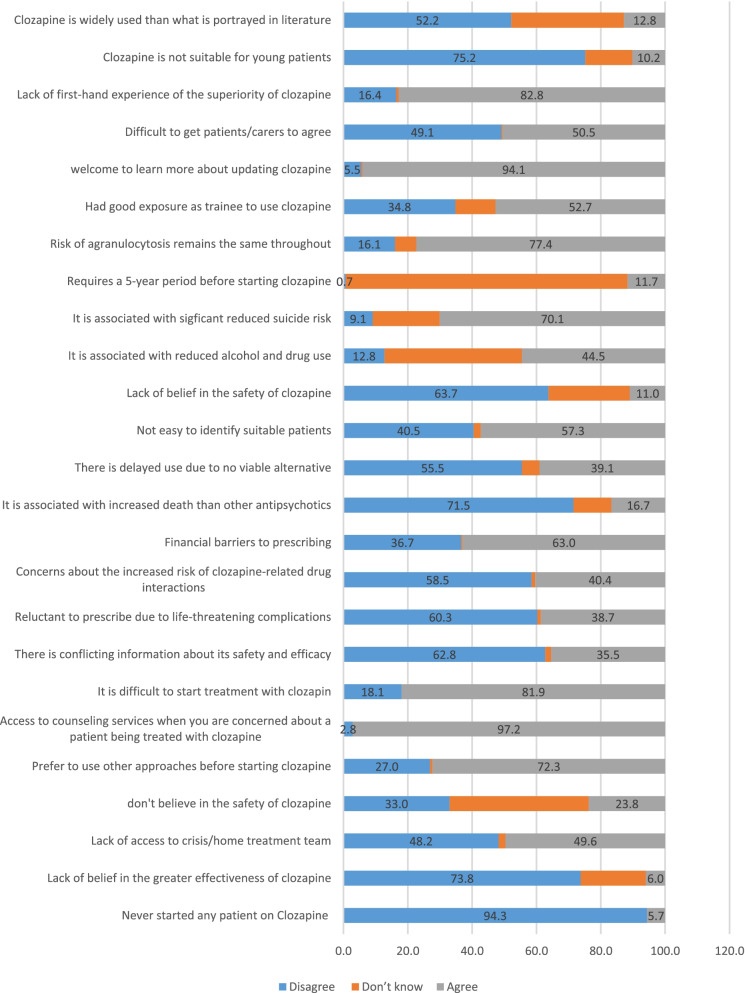
Knowledge, attitude, and experience of using clozapine

Approximately 42% had no opinion, and about 70% believed that clozapine was associated with a reduced risk of suicide. Although about 53% of respondents believed they had good clinical experience regarding clozapine prescription and side effects management, approximately 77% of psychiatrists agreed that the risk of developing agranulocytosis as long as a person is treated with clozapine is still high. Most participants believed they needed more training and updates about using clozapine. About half of the participants found it challenging to get patients or caregivers to agree to initiate clozapine. About 83% said they had no first-hand experience of the superiority of clozapine treatment. Two-thirds of the participants believed that clozapine was suitable for young patients. More than half of the participants disagreed that clozapine is prescribed more than depicted in the articles, and only about 13% agreed (Fig. 1).

### Association between academic position and knowledge and attitude

Psychiatrists with an academic position reported that they had access to appropriate facilities for the control and management of patients treated with clozapine (*p* < 0.05) and believed in the safety of the clozapine (*p* < 0.05) (Table 3). Psychiatrists with no academic position believed that it is not easy to quickly diagnose patients who benefit from changing their medication to clozapine (*p* < 0.05) and had no first-hand experience of the superiority of clozapine treatment (*p* < 0.01) (Table 3).

**Table 3:** Association between knowledge and attitude and academic position

Questions	Had an academic position			No academic position			***p***-value
	Disagree	Don’t know	Agree	Disagree	Don’t know	Agree	
Never started any patient on Clozapine	48 (100)	0	0	210 92.9)	0	16 (7.1)	0.084*
Lack of belief in the greater effectiveness of clozapine	35 (72.9)	10 (20.8)	3 (6.3)	166 (73.5)	47 (20.8)	13 (5.8)	0.991**
Lack of access to crisis/home treatment team	18 (37.5)	3 (6.5)	27 (56. 5)	113 (50.0)	3 (1.3)	110 (48. 7)	0.047
don’t believe in the safety of clozapine	9 (18.8)	23 (47.9)	16 (33.3)	81 (35.8)	96 (42.5)	49 (21.7)	0.048
I prefer to use other approaches before starting clozapine	18 (37.5)	0	30 (62.5)	57 (25.2)	2 (0.9)	167 (73.9)	0.187*
Access to counseling services	0	0	48 (100)	8 (3.6)	0	217 (96.4)	0.358
It is difficult to start treatment with clozapine	7 (14.6)	0	41 (85.4)	41 (18.1)	0	185 (81.7)	0.678*
There is conflicting information about its safety and efficacy	36 (75.0)	0	12 (25.0)	137 (60.6)	5 (2.2)	84 (37.2)	0.144*
Reluctant to prescribe due to life-threatening complications	30 (62.5)	0	18 (37.5)	134 (59.3)	3 (1.3)	89 (39.4)	0.928*
Concerns about the increased risk of clozapine-related drug interactions	31 (64.6)	0	17 (35.4)	130 (57.5)	3 (1.3)	93 (41.2)	0.730*
Financial barriers to prescribing	21 (43.8)	1 (2.1)	26 (54.2)	78 34.5)	0	148 (65.5)	0.059*
It is associated with increased death than other antipsychotics	35 (72.9)	3 (6.3)	10 (20.8)	160 (70.8)	29 (12.8)	37 (16.4)	0.377
There is delayed use due to no viable alternative	29 (61.7)	2 (4.3)	16 (34.0)	120 (54.8)	13 (5.9)	86 (39.3)	0.671
Not easy to identify suitable patients	23 (48.9)	3 (6.4)	21 (44.7)	85 (38.8)	3 (1.4)	131 (59.8)	0.033
Lack of belief in the safety of clozapine	31 (65.9)	8 (17.0)	8 (17.0)	139 (63.5)	59 (26.9)	21 (9.6)	0.173
It is associated with reduced alcohol and drug use	9 (19.2)	17 (36.2)	21 (44.7)	25 (11.4)	98 (44.8)	96 (43.8)	0.290
It is associated with Signiant reduced suicide risk	6 (12.8)	7 (14.9)	34 (72.3)	19 (8.7)	49 (22.4)	151 (68.9)	0.410
Requires 5 years before starting clozapine	2 (4.3)	37 (78.7)	8 (17.0)	0	196 (89.5)	23 (10.5)	0.012
The risk of agranulocytosis remains the same throughout	8 (17.0)	4 (8.5)	35 (74.5)	34 (15.5)	13 (5.9)	172 (78.5)	0.764
Had good exposure as a trainee to use clozapine	11 (23.9)	5 (10.9)	30 (65.2)	81 (36.9)	27 (12.3)	111 (50.7)	0.178
Welcome to learn more about updating clozapine	3 (6.4)	1 (2.1)	43 (91.5)	11 (5.1)	0	207 (94.9)	0.146*
Challenging to get patients/careers to agree	23 (48.9)	1 (2.1)	23 (48.9)	109 (49.8)	0	110 (50.2)	0.242*
Lack of first-hand experience of the superiority of clozapine	17 (36.2)	0	30 (63.8)	28 (12.8)	2 (0.9)	189 (86.3)	0.001*
Clozapine is not suitable for young patients	37 (78.7)	3 (6.4)	7 (14. 9)	164 (74.9)	34 (15.5)	21 (9.6)	0.162
Clozapine is more widely used than what is portrayed in literature	29 (61.7)	12 (25.5)	6 (12.8)	110 (50.2)	80 (36.5)	29 (13.2)	0.311

*Fisher exact test and chi-square for the rest without*

### Association between work experience and knowledge, and attitude

Work experience of 15 years and more was significantly associated with higher prescription of clozapine (*p* < 0.0001), belief in greater effectiveness of clozapine (*p* < 0.0001), and its safety (p < 0.0001). Psychiatrists with work experience of less than 15 years agreed more with difficulty in initiating clozapine treatment (*p* < 0.0001), reluctant to prescribe clozapine due to its life-threatening side effects (*p* < 0.0001) and agreed more with concerns about increasing the likelihood of drug interactions (*p* < 0.05). In addition, they were more agreed with the view that clozapine increased the risk of death compared to other antipsychotic drugs (*p* < 0.0001) and felt “clozapine should be delayed due to the lack of other suitable alternatives (*p* < 0.0001) (Table 4).

**Table 4:** Association between knowledge and attitude and work experience

Questions	less than 15 years			15 years and more			***p***-value
	Disagree	Don’t know	Agree	Disagree	Don’t know	Agree	
Never started any patient on lozapine	130 (90.3)	0	14 (9.7)	132 (100)	0	0	< 0.0001*
Lack of belief in the greater effectiveness of clozapine	93 (64.6)	39 (27.1)	12 (8.3)	112 (84.8)	18 (13.6)	2 (1.5)	< 0.0001*
Lack of access to crisis/home treatment team	65 (45.1)	4 (2.8)	75 (52.1)	66 (50.0)	2 (1.5)	64 (48.5)	0.611
don’t believe in the safety of clozapine	32 (22.2)	76 (52.8)	36 (25.0)	60 (45. 5)	43 (32.6)	29 (21.9)	< 0.0001
I prefer to use other approaches before starting clozapine	42 (29.2)	2 (1.4)	100 (69.4)	34 (25.8)	0	98 (74.2)	0.381*
Access to counseling services	6 (4.2)	0	138 (95.8)	2 (1.5)	0	129 (98.5)	< 0.174
It is difficult to start treatment with clozapine	15 (10.4)	0	129 (89.6)	36 (27.3)	0	96 (72.7)	< 0.0001
There is conflicting information about its safety and efficacy	84 (58.3)	2 (1.4)	58 (40.3)	91 (68.9)	3 (2.3)	38 (28.8)	< 0.132
Reluctant to prescribe due to life-threatening complications	73 (50.7)	0	71 (49.3)	95 (71.9)	3 (2.3)	34 (25.8)	< 0.0001*
Concerns about the increased risk of clozapine-related drug interactions	76 (52.8)	1 (0.7)	67 (46.5)	87 (65.9)	2 (1.5)	43 (32.6)	< 0.037*
Financial barriers to prescribing	50 (34.7)	1 (0.7)	93 (64.6)	53 (40.5)	0	78 (59.5)	0.383
It is associated with increased death than other antipsychotics	88 (61.1)	25 (17.4)	31 (21.5)	110 (83.9)	7 (5.3)	14 (10.7)	< 0.0001
There is delayed use due to no viable alternative	63 (45.7)	8 (5.8)	67 (48.6)	88 (67.7)	6 (4.6)	36 (27.7)	0.001*
Not easy to identify suitable patients	50 (36.2)	5 (3.6)	83 (60.1)	60 (46.2)	1 (0.8)	69 (53.1)	0.1
Lack of belief in the safety of clozapine	72 (52.6)	45 (32.9)	20 (14.6)	99 (76.2)	22 (16.9)	9 (6.9)	< 0.0001
It is associated with reduced alcohol and drug use	18 (13.0)	84 (60.9)	36 (26.1)	16 (12.3)	30 (23.1)	84 (64.6)	< 0.0001
It is associated with Signiant reduced suicide risk	12 (8.7)	41 (29.7)	85 (61.6)	13 (10.0)	14 (10.8)	103 (79.2)	0.001
Requires 5 years before starting clozapine	1 (0.7)	127 (92.0)	10 (7.3)	1 (0.8)	108 (83.1)	21 (16.2)	0.04*
The risk of agranulocytosis remains the same throughout	23 (16.7)	12 (8.7)	103 (74.6)	21 (16.2)	6 (4.6)	103 (79.2)	0.396
I had good exposure as a trainee to using clozapine	68 (49.3)	24 (17.4)	46 (33.3)	24 (18.6)	9 (6.9)	96 (74.4)	< 0.0001*
Welcome to learn more about updating clozapine	9 (6.6)	0	128 (93.4)	6 (4.6)	1 (0.8)	123 (94.6)	0.514
Challenging to get patients/careers to agree	50 (36.5)	0	87 (63.5)	83 (63.9)	1 (0.8)	46 (35.4)	< 0.0001*
Lack of first-hand experience of the superiority of clozapine	15 (10.9)	2 (1.5)	121 (87.7)	30 (23.1)	0	100 (76.9)	< 0.008
Clozapine is not suitable for young patients	97 (70.3)	23 (16.7)	18 (13.0)	105 (80.8)	15 (11.5)	10 (7.7)	0.127
Clozapine is more widely used than what is portrayed in literature	50 (36.2)	70 (50.7)	18 (13.0)	90 (69.2)	25 (19.2)	15 (11.5)	< 0.0001

*Fisher exact test and chi-square for the rest without *

Psychiatrists with work experience of 15 years and more agreed more with the safety of patients starting treatment with clozapine (*p* < 0.0001). They believed that clozapine was associated with a reduction in alcohol and drug use and the risk of suicide among patients treated (*p* < 0.0001 and *p* < 0.005, respectively) (Table 4).

Psychiatrists with work experience of 15 years and more agreed more with and had good clinical experience with clozapine and its side effects (*p* < 0.0001) but disagreed more that clozapine is prescribed more than what is depicted in the articles (*p* < 0.0001). On the other hand, psychiatrists with work experience of less than 15 years agreed more with difficulty in getting patients or caregivers to agree to initiate clozapine and had no first-hand experience of the superiority of clozapine treatment (*p* < 0.05) (Table 4).

## Discussion

This is the first nationwide study investigating clozapine prescription for patients with TRS from Iranian psychiatrists’ perspective. Using a questionnaire, we assessed the attitude and knowledge of 282 psychiatrists working in different parts of Iran. Since, in Iran, psychiatrists have the leading role in decision-making to prescribe clozapine for patients with TRS, the results will be important to help assess the status of clozapine prescription in Iran. Our results showed about 93% of respondents prescribed clozapine for their patients in their work experience. In contrast, about 70% of them had less than five patients under clozapine treatment, and 90% reported that there is no dedicated physician in their organization. The results suggest that despite commonly reported concern about serious side effects of clozapine, prescription of clozapine is not common among Iranian psychiatrists. The issue is promising for the treatment of patients with TRS.

On the other hand, a low number of undertreated patients (less than 5 patients) in more than of 2/3of them may raise a concern about the management of under-treated patients, i.e., while clozapine is being started, patients and families may refuse treatment continuation due to various reasons such as lack of access to do a routine lab test. Physicians may discontinue treatment due to the serious side effects of clozapine (as included in the result, less than half of the participants experienced serious side effects in their patients.). Consequently, under-use of clozapine may emerge. The frequency of clozapine prescription and less than 5 under-treated patients has been reported (78 and 56%, respectively) in Leung et al.’s study [30]. Strategies to enhance patients’ adherence, including follow-up teams and increasing patients’ and families’ literacy about treatment with clozapine, can effectively increase the number of under-treated patients and clozapine prescriptions.

Our results also showed that in some aspects, there is relatively good knowledge and a positive attitude toward using clozapine among Iranian psychiatrists. These include starting clozapine for the eligible patients (94%), believing in more effectiveness of clozapine (74%), welcome to learn more about clozapine updating (94.1%), belief in reducing the risk of suicide by clozapine (70.1%), access to counseling service for resolving concern about under-treated patients (97%), disagreement with the unsuitability of clozapine for young patients (75.2%), and disagreement with increasing risk of death with clozapine compared other antipsychotics drugs. In a similar study by Tungaraza and Farooq, these percentages have been reported lower in some cases [21]. Perhaps the two studies’ time interval of about 6 years (2013–2019) can justify this difference. At the interval, more evidence to support the efficacy and safety of clozapine has been published, which can be effective on attitude toward using clozapine.

On the other hand, use of different strategies before initiating clozapine (73%), having difficulty in initiating clozapine (81.2%), delay in initiating clozapine due to financial issues (63%), difficulty in diagnosis of suitable patients for clozapine treatment (60%), believe the high risk of developing agranulocytosis as long as a person treated with clozapine (77%), and lack of the firsthand experience of the superiority of clozapine (88%) were other results which addressed to clozapine use related concerns among Iranian psychiatrist to use of clozapine. The concerns may lead to underutilization. Therefore, addressing these concerns using different strategies seems necessary. Documented reports on follow-up of patients treated with clozapine and periodic continuous education programs about updated guidelines on clozapine use are the strategies to reduce the concerns. The concerns were also reported in the previous studies in this area. Kelly et al., in their report, addressed this issue. They also provided several recommendations for increasing clozapine use for different groups involved in clozapine use (including psychiatric residency/fellowship programs, academic health centers, acute care and psychiatric hospitals, academic scientists, and..) [31]. It seems using the experience can be helpful for Iranian psychiatrists who want to overcome clozapine-related concerns.

As mentioned earlier, our results showed an association between having an academic position and access to the facility to control and manage patients treated with clozapine and believing in the safety of clozapine (*p* < 0.05). On the other hand, participants with no academic position have difficulty diagnosing eligible patients for starting clozapine(p < 0.05). They also had no firsthand experience with the superiority of clozapine(*p* < 0.01). Having an academic position can allow the psychiatrist easy access to training hospitals. There is the possibility of collaborating with other professionals, such as clinical pharmacologists and nursing staff, which facilitates diagnosing and managing patients who should undergo clozapine treatment. To the best of our knowledge, there is no outpatient clozapine treatment in Iran. The interprofessional practice model to improve and optimize clozapine prescribing has been emphasized by Warnez and Alessi-Severini [32].

Finally, our results showed that more work experience (15 years and more) had association with a higher prescription of clozapine, belief in greater effectiveness of clozapine, its safety, and having good clinical experience with clozapine and its side effects (*p* < 0.01), while low work experience (less than 15 years) had an association with difficulty to get patients or caregivers to agree to initiate clozapine, and had no first-hand experience of the superiority of clozapine treatment (*p* < 0.05). Work experience can play an important role in prescribing clozapine from some aspects. First, in Iranian culture, the experience is an advantage for physicians to get patients’ trust and prevent dropouts [33]. Secondly, work experience can increase the number of patients who underwent clozapine treatment, enhancing the psychiatrist’s practical ability and basic knowledge about clozapine. Finally, work experience is usually associated with successful treatment experience, which can decrease the concerns of young physicians. The association between longer clinical experience and more willingness to prescribe clozapine has been reported in studies conducted in both developing [14, 34] and developed countries [35]. Collaboration between young psychiatrists and psychiatrists with longer experience can help develop a positive attitude toward clozapine prescription.

## Limitations

The study has limitations that should be mentioned. Our study was a cross-sectional study conducted among Iranian psychiatrists registered by IPA; we did not have access to those who did not register, and we could not assess their knowledge and attitude, so the generalization of the results to all Iranian psychiatrists is not straightforward. Families and caregivers of patients with TRS have important role in clozapine use. In this study, we did not include them. Further research to overcome the limitations is recommended.

## Conclusions

In this study, we found that most Iranian psychiatrists had a good self-perception knowledge about the efficacy of clozapine for patients with TRS. Still, concerns about serious side effects may discourage them from starting clozapine. Psychiatrists with longer work experience and academic positions had a more optimistic attitude toward clozapine use than the younger psychiatrists with no academic position. Implementing continuous educational programs and leading treatment processes by experienced psychiatrists can help develop an optimistic view of young psychiatrists.

## Data Availability

The datasets used and/or analyzed during the current study are available in the Persian language in: https://digit.kums.ac.ir/s/3008412.html, but due to the confidentiality of data, it would be available from the corresponding author upon reasonable request.
